# Evaluation of Fentanyl Disposition and Effects in Newborn Piglets as an Experimental Model for Human Neonates

**DOI:** 10.1371/journal.pone.0090728

**Published:** 2014-03-04

**Authors:** Carmen Rey-Santano, Victoria Mielgo, Adolfo Valls-i-Soler, Esther Encinas, John C. Lukas, Valvanera Vozmediano, Elena Suárez

**Affiliations:** 1 Experimental Neonatal Physiology Unit, BioCruces Health Research Institute, Cruces University Hospital, Barakaldo, Bizkaia, Spain; 2 Department of Pharmacology, Faculty of Medicine, University of the Basque Country, UPV/EHU, Leioa, Bizkaia, Spain; 3 Drug Modeling and Consulting, Dynakin SL, Derio, Bizkaia, Spain; The Ohio State Unversity, United States of America

## Abstract

**Background:**

Fentanyl is widely used off-label in NICU. Our aim was to investigate its cerebral, cardiovascular and pulmonary effects as well as pharmacokinetics in an experimental model for neonates.

**Methods:**

Fentanyl (5 µg/kg bolus immediately followed by a 90 minute infusion of 3 µg/kg/h) was administered to six mechanically ventilated newborn piglets. Cardiovascular, ventilation, pulmonary and oxygenation indexes as well as brain activity were monitored from T = 0 up to the end of experiments (T = 225–300 min). Also plasma samples for quantification of fentanyl were drawn.

**Results:**

A “reliable degree of sedation” was observed up to T = 210–240 min, consistent with the selected dosing regimen and the observed fentanyl plasma levels. Unlike cardiovascular parameters, which were unmodified except for an increasing trend in heart rate, some of the ventilation and oxygenation indexes as well as brain activity were significantly altered. The pulmonary and brain effects of fentanyl were mostly recovered from T = 210 min to the end of experiment.

**Conclusion:**

The newborn piglet was shown to be a suitable experimental model for studying fentanyl disposition as well as respiratory and cardiovascular effects in human neonates. Therefore, it could be extremely useful for further investigating the drug behaviour under pathophysiological conditions.

## Introduction

Fentanyl is a potent synthetic opioid which is increasingly used in neonatal intensive care unit (NICU) and pediatric intensive care unit (PICU) as postoperative analgesic, and as a sedative for patients requiring mechanical ventilation. Fentanyl is frequently preferred over morphine because of its wider therapeutic index, lack of histamine release and limited adverse effects on pulmonary and hemodynamic function [1]. Moreover, the higher lipid solubility and less complex receptor binding of fentanyl as compared to morphine allow a rapid penetration of the blood-brain barrier and a rapid onset of action [2]. Its use in pediatric critical care has significantly increased for the past 20 years; however, it remains one of the many medications which have not been properly tested in this population [3].

In clinical practice, pediatric dosing regimens are often empirically derived from those employed in adults on a body weight basis. However, this procedure is not fully justifiable because ontogenic maturation processes related to drug pharmacokinetics and pharmacodynamics (PK/PD) are not necessarily always body weight correlates. Specifically for fentanyl, the impact of ontogeny is considered to be more outstanding regarding PK, as the drug is subject to hepatic biotransformation [4] and highly bound to plasma proteins, primarily α_1_-acid glycoprotein (AAG) [5], [6]. In this sense, a maturation physiology based predictive PK/PD model for fentanyl in neonatal care was built, starting from the drug disposition in adults and including those developmental changes associated to growth (enzyme activity, organ weights, blood flows, AAG, etc.) [7].

The relevance of this type of models lies in that it allows minimizing the chance of overdose-associated adverse effects (e.g. skeletal muscle rigidity, hypoxia, desaturations, hypercapnia and limited haemodynamic imbalance) [8]–[13], which might be more severe in the youngest patients. In sick newborn infants with impaired cerebral autoregulation, those adverse effects might in turn cause fluctuations in cerebral blood flow (CBF) and electroencephalogram abnormalities, which are risk factors for brain injury and long-term disability. Nonetheless, virtually no data concerning the effects of fentanyl administration on cerebral activity, metabolism and circulation is available for neonates.

Given the difficulties of doing pharmacological research in the neonatal population, the performance of experimental studies in suitable animal species is also a common practice, often in addition or as a complement to previously developed theoretical models. In this respect, pigs have been used as experimental animals for a long time, because many of their anatomical and physiological characteristics more closely resemble those of humans than other non-primate species [14], [15]. Specifically, the newborn piglet is a representative model for the cardiovascular physiologic development of neonates [13]. Furthermore, similarities in porcine drug metabolizing enzymes suggest that pigs may be the most suitable animal model for drug biotransformation studies. In this sense, activity of the most important CYP isoform in humans, CYP3A4, also responsible for hepatic fentanyl metabolism, is present in pigs with comparable levels and activities, as opposed to other routinely used experimental animals [14], [16]. Similarly, for some drugs, the differences observed between juvenile and adult pig PK were deemed as consistent with ontogenic changes reported for human PK [15].

Although direct extrapolation of animal data to humans is not appropriate, we hypothesized that preclinical evidence from the newborn piglet model elucidating the potential impact of pharmacotherapy on the developing brain forms could be an essential bridge to human studies and informed clinical practice. The purpose of this study was to investigate the main cardiovascular and pulmonary effects as well as the PK profile of fentanyl when administered as the only narcotic agent in healthy mechanically ventilated newborn piglets. Also, the brain effect of fentanyl was tested by using the two devices available for actual bedside studies in neonates: near-infrared spectroscopy (NIRS), a non-invasive optical technique for assessing circulation and oxygenation in brain, together with amplitude-integrated electroencephalography (aEEG). As a second objective, the study was to provide further confirmatory evidence of the previously developed physiology based predictive PK/PD model for fentanyl in neonatal care [7].

## Methods

A prospective study was performed in eight newborn piglets (2–4 days, 1.7±0.2 kg) of either sex. This study was carried out in strict accordance with the recommendations in the Guide for the Care and Use of Laboratory Animals. The experimental protocol met European and Spanish regulations for protection of experimental animals (86/609/EEC and RD 1201/2005) and was approved by the Ethical Committee for Animal Welfare of the Cruces University Hospital.

### Surgical preparation

The animals were sedated with i.m. ketamine (15 mg/kg) and diazepam (2 mg/kg) administration. A face mask was applied and animals were anesthetized with 1.5%–2% of sevoflurane and oxygen.

A tracheotomy was performed; a tracheal tube (4.0 mmID) was inserted and connected to an inhalator anaesthesia system. Animals were then positive pressure ventilated (VIP Bird, Bird Products Corp., Palm Springs, CA) with the following initial settings: fraction of inspired oxygen (F_iO2_) = 0.25, respiratory frequency (f_R_) = 20 breaths/min, positive end-expiratory pressure (PEEP) = 3 cmH_2_O and positive inspiratory pressure (PIP) = 9 cmH_2_O. Deviations from acceptable blood gases values (PaO_2_ 90–110 mmHg, PaCO_2_ 35–45 mmHg and pH 7.35–7.45) were corrected by changing ventilator parameters and/or by adding sodium bicarbonate as needed.

A thermodilution arterial catheter (5Fr, PiCCO Plus, Pulsion, München, Germany) was inserted into the femoral artery to monitor mean arterial blood pressure (MABP), heart rate (HR) and cardiac output (CO) as well as to obtain blood samples for gas analysis and subsequent determination of fentanyl plasma concentration. Also, a 5 Fr three-lumen catheter was inserted into the internal jugular vein to allow injection of cold saline, measurement of central venous pressure (CVP), maintenance of fluids (5 ml/kg/h) and fentanyl infusion. Jugular vein catheterization was used to infuse vecuronium (3 mg/kg bolus every 1 hour until the end of fentanyl infusion) to avoid breathing and muscle movements that may distort the two-channel bedside aEEG (BRM2; BrainZ Instruments, Auckland, New Zealand), NIRS system (NIRO-200; Hamamatsu Photonics KK, Joko-Cho, Japan) or lung mechanics data. Lung tidal volume and pulmonary dynamic compliance (C_dyn_) were continuously monitored by computerized pneumatocography (Mod. M2780A, Philips Medizin Systeme, Boblingen, Germany).

### A pilot study in two initial piglets

Testing in the two first piglets was conceived as a pilot study aimed to refine, if necessary, the dosing schedule, the sampling times and/or the duration of the experiment. These animals were administered i.v. fentanyl bolus dose of 4.8 µg/kg and a simultaneously starting 30 minute infusion at a rate of 6 µg/kg/h, while trials were set to be stopped at 90 minutes after infusion discontinuation. The mentioned regimen was selected based on the PK parameters available in the literature for fentanyl in newborn piglets [13] to achieve a concentration at steady state (Css) of 5 ng/ml. In the absence of specific data for piglets, the concentration reported to produce 50% maximum sedation effect (EC_50_) in human newborns (i.e. 3 ng/ml) [7] was assumed to be also true for this species, so the aim was to preclude any possibility of alertness in those animals, in line with the shown ethical concern.

Observations from the pilot phase were compared with the estimates provided by the ontogeny based PK/PD model in the neonate [7] and served to redefine the study protocol ahead of the experimentation in the remaining planned animals. Only the results corresponding to the final protocol (n = 6) were included in the overall analysis.

### Experimental protocol and acquisition of physiological variables

After surgical preparation, baseline conditions (Basal1) were registered, following which the inhalator anaesthesia system was stopped. 5 min later, time considered enough to eliminate the sevoflurane anesthetic effects, basal values were checked again (Basal2). Immediately afterwards, animals received an i.v. fentanyl bolus dose (5 µg/kg) and a simultaneously starting continuous i.v. infusion of fentanyl at a rate of 3 µg/kg/h for 90 min.

Arterial blood samples for gas analysis (pH, Pa_O2_, Pa_CO2_, and arterial oxygen saturation: S_aO2_) and 1 ml for subsequent quantification of fentanyl plasma levels were withdrawn at the following timepoints: Basal 1, Basal 2, immediately after bolus administration (Bolus), 1, 10, 30, 90, 95, 120 and 180 min after the start of the infusion and then every 30 min until the end of the experiment.

Also, at each timepoint cardiovascular parameters (MABP, HR, and CO), ventilator parameters (PIP, PEEP, f_R_ and F_iO2_) and lung mechanics were registered. Moreover, OI: mean airway pressure (cmH_2_O)*F_iO2_/P_aO2_ (mmHg) and ventilator efficiency index (VEI): 3800/PIP-PEEP*f_R_ (breath/min)*P_aCO2_ (mmHg) were also calculated.

The end of the experiment was arbitrarily established as the moment when application of a painful stimuli resulted in a significant HR increase (≥15 beats/min) and/or an increment in the aEEG amplitude activity greater than 30% over the previous measurement. At the end of each experiment, the piglets were sacrificed with an overdose of sevoflurane, vecuronium and potassium chloride.

### Neurophysiological assessment

Change in cerebral perfusion-oxygenation was assessed using NIRS system. The sensor was placed on the skull in the midline fronto-parietal area. Tissue oxygen index (TOI) and variations in tissue hemoglobin index (THI) were continuously monitored. TOI represents the tissue oxygen saturation and is measured in percent; it was used to calculate the fractional tissue oxygen extraction (FTOE) [17]: FTOE = (S_aO2_-TOI)/S_aO2_. THI is an absolute figure of the total Hb content in brain; its changes were used to estimate changes in cerebral blood volume [18]. The cerebral intravascular oxygenation (CIO), equivalent to the difference between oxyhemoglobin and deoxyhemoglobin, was used as surrogate of CBF changes [19].

Brain activity was monitored using a two-channel bedside aEEG monitor with five needle electrodes. The aEEG background activity and the aEEG amplitude were measured. NIRS and aEEG parameters were continuously recorded throughout the experimental period.

### Handling and measurement of fentanyl in plasma samples

Arterial blood samples for analysis of plasma fentanyl concentrations were collected in EDTA tubes, and kept on ice until their immediate centrifugation at 3000 rpm for 10 minutes. Once separated, plasma was transferred to cryovials and initially frozen at –20°C, followed by a subsequent storage at –80°C until analyzed. Plasma fentanyl levels were measured by a selective and sensitive high-performance liquid chromatography-tandem mass spectrometry (HPLC/MS-MS) method. The assay, specific for fentanyl and not accounting for its metabolites, had a sensitivity of 0.2 ng/ml, as represented by the lower limit of quantification (LLOQ). Intra and interassay coefficients of variation did not exceed 5% and 15%, respectively.

### Evaluation of the pharmacokinetic variables

Noncompartmental pharmacokinetic analysis (NCA) was performed on the plasma concentration data using Phoenix^TM^ WinNonLin (version 6.1, Pharsight Corporation, St. Louis, MO, USA). Solely those plasma samples with fentanyl levels above the LLOQ were considered for the analysis. Peak plasma concentration (C_max_), time to reach C_max_ (t_max_), last quantifiable concentration (C_last_) and the corresponding timepoint for C_last_ (T_last_) were obtained directly from the measured concentration-time curves. The area under the plasma concentration-time curve from time 0 to the last measurable concentration (AUC_0-t_) was calculated by the trapezoidal formula. Systemic clearance (CL) was calculated from the ratio between the infusion dose and the exposure (AUC_0-t_).

Furthermore, an exploratory analysis aimed to elucidate the relationship between these PK variables and the observed effect was performed. Evaluation was focused on the degree of sedation, represented by aEEG measured brain activity, as the main effect targeted by drug administration.

### Determination of plasma AAG as a potential source of PK variability

In an attempt to partially account for interindividual PK variability (IIV) on fentanyl disposition and to assess if plasma levels of this acute phase protein were modified by the experimental procedure, concentrations of AAG in plasma samples (basal, 90 min and last timepoint) from six piglets were determined using a Porcine AAG ELISA kit (AMS Biotechnology Ltd., Oxfordshire, UK). Analyses were performed by duplicate and the absorbance was measured in a microplate reader (POLARstar, BMG, Ortenberg, Germany) at 450 nm. For further comparison, AAG was also quantified in blank plasma samples from additional newborn piglets acting as controls.

### Statistical Analysis

Data was analyzed using JMP statistical discovery software (version 8, SAS Institute Inc., North Carolina, USA). Comparison of measured values before and after fentanyl administration was assessed by t test of paired means. Simple linear regression analysis was done to assess the relationship between plasma AAG levels and fentanyl exposure. A p< 0.05 was considered statistically significant. Values are expressed as mean ± SD.

## Results

### Observations from the pilot study (n = 2)

NCA of the plasma profiles in two initial piglets revealed an average CL of 0.022 L/min, which is of the same order of magnitude as the one calculated by the above mentioned ontogeny predictive model for human newborns (i.e., 0.028 L/min) [7]. Based on this fact, fentanyl disposition in piglets was assumed to be comparable to human newborns and, consequently, the protocol was amended to adapt the dosing schedule from that usually employed in the NICU for sedation (i.e., a 2–5 µg/kg bolus immediately followed by an infusion of 1–3 µg/kg/h). Namely, the highest dose level in the range was found suitable, via simulation, to provide a Css of roughly 3 ng/ml, which was deemed as an appropriate target concentration in view of the somewhat excessive degree of sedation attained for a Css of 5 ng/ml, as per the observed aEEG (data not shown). In addition, the observation that animals were sufficiently controlled when receiving fentanyl as the sole narcotic agent encouraged to enlarge both the infusion duration and the observation period after infusion discontinuation.

### Overall data corresponding to the final protocol (n = 6)

#### Physiological variables

Baseline conditions were consistent with those previously reported for newborn piglets [18]. The animals remained stable throughout the study and presented a reliable degree of sedation for 120–150 min after fentanyl infusion; only some agitation was noted at the end of the experiment. In this sense, the study period upon completion of fentanyl administration was 150 min in two of the six animals while it was ≥180 min in the remaining.

**Figure 1:**
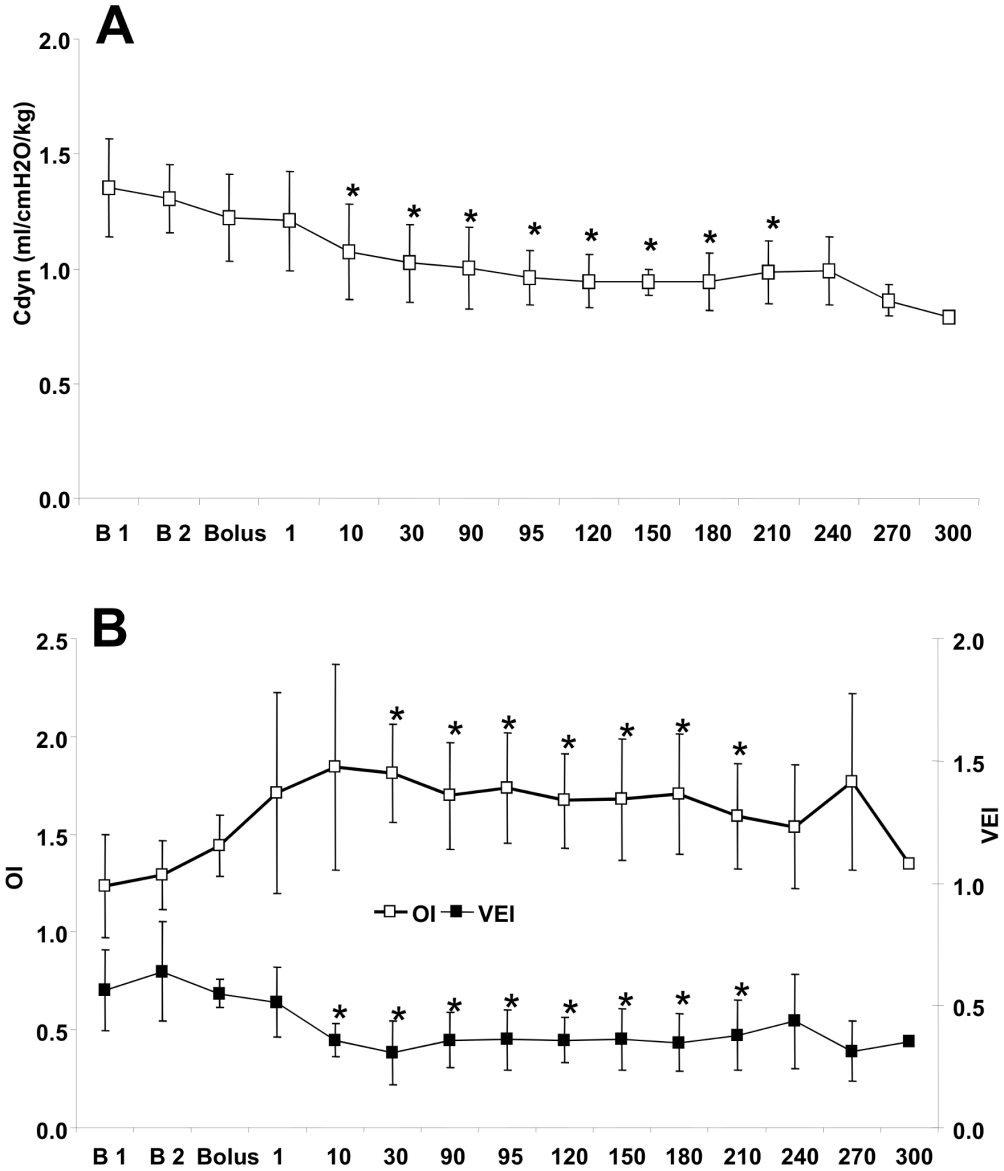
Pulmonary effects of fentanyl administration in newborn piglets maintained on mechanical ventilation. A- Mean dynamic compliance, C_dyn_, B- mean oxygenation index, OI, (white squares) and mean ventilator efficiency index,VEI, (black squares). Values are expressed as mean±SD. Normal values: OI<10; VEI>0.3; C_dyn_: 0.8–1.5 ml/cmH_2_O/kg. The letter “B1” indicates the baseline measurement during inhaler anesthesia and letter “B2” indicates the baseline measurement after elimination of inhaler anaesthesia. *p <0.05 vs. baseline 2 level.

Arterial pH and Pa_CO2_ transiently changed (Table 1) due to a significant decrease in C_dyn_ (Figure 1A) observed after fentanyl bolus administration. Due to changes performed in the ventilatory setting of mechanical ventilator, gas exchange was maintained within normal ranges for all the study. However, VEI and OI were significantly altered during the first minute and during initial 10 min, respectively, following fentanyl bolus administration (Figure 1B). Those parameters remained unchanged for 120 min after completion of the fentanyl infusion. At the end of the experiment (last 30 min in each animal procedure) the gas exchange (Table 1), C_dyn_ (Figure 1A) and ventilation indexes (Figure 1B) showed great variations due to the agitation perceived in animals.

**Table 1:** Gas exchange and hemodynamic parameters in newborn piglets treated with fentanyl.

	pH	P_aCO2_ (mmHg)	MABP (mmHg)	HR (beats/min)	CO (l/min)	CVP (mmHg)
B1	7.41±0.06	36±8	62±10	158±27	0.5±0.1	4±1
B2	7.37±0.05	42±4	77±6	182±29	0.6±0,1	3±1
Bolus	7.37±0.04	47±5*	76±8	179±37	0.6±0.1	3±1
1 min	7.31±0.06*	49±4*	72±8	175±37	0.6±0.1	3±1
10 min	7.33±0.06	45±6	80±6	186±47	0.6±0.2	3±1
30 min	7.34±0.10	44±7	86±9	212±63	0.5±0.1	3±1
90 min	7.38±0.04	39±8	85±7	235±54*	0.6±0.1	4±1
95 min	7.39±0.05	38±6	83±5	241±54*	0.6±0.1	3±2
120 min	7.39±0.04	39±6	78±10	245±42*	0.6±0.1	4±1
150 min	7.38±0.04	40±6	83±8*	259±45*	0.7±0.1	3±2
180 min	7.37±0,04	41±6	82±7	254±40*	0.6±0	3±2
210 min	7.41±0,04	38±9	79±12	254±35*	0,6±0.1	3±1
240 min	7.45±0.06	34±8	75±8	250±48*	0.6±0,1	3±1
270 min	7.42±0.06	40±11	81±12	254±44*	0.5±0,1	3±1
300 min	7.43	30	70	270*	0.6	4

Normal values (aprox): pH: 7.3–7.45; Pa_CO2_: 35–45 mmHg; MABP: 60–85 mmHg; HR: 155–195 beats/min;CO: 0.4–0.7 l/min; CVP: 3–6 mmHg.

B1: baseline 1; B2: baseline 2; MABP: mean arterial blood pressure; HR: heart rate; CO: cardiac output; CVP: central venous pressure. Values are expressed as mean±SD. *p<0.05 vs. B2.

Cardiovascular response is shown in Table 1. Compared to pre-treatment values (Basal 2), HR increased from T = 90 min to the end of the experiment with no significant change in MABP, CO or CVP.

#### Neurophysiological assessment

**Figure 2:**
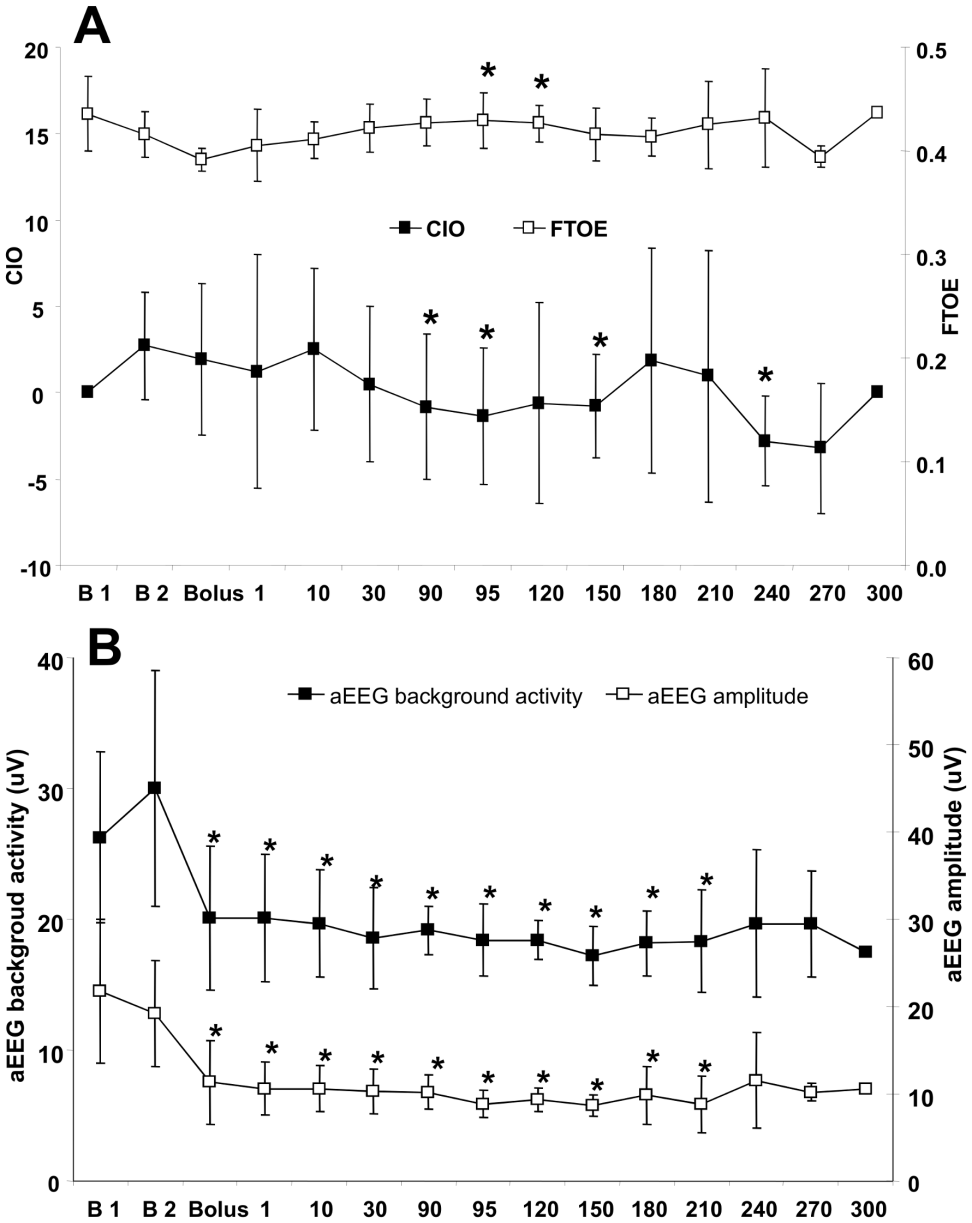
Evaluation of neurophysiological assessment in newborn piglets treated with fentanyl. A-Cerebral perfusion-oxygenation using NIRS system. Mean cerebral intravascular oxygenation, CIO, (black squares) and mean fractional tissue oxygen extraction, FTOE, (white squares). B- Brain activity using aEEG system. Normal values: FTOE: 0.3–0.6; CIO: (–10) –10. The letter “B1” indicates the baseline measurement during inhaler anesthesia and letter “B2” indicates the baseline measurement after elimination of inhaler anaesthesia. Mean aEEG background activity (black squares) and mean aEEG amplitude (white squares). *p <0.05 vs. baseline 2 level.

Fentanyl administration produced a continuous decrease of CIO over time, reaching statistical significance at the end of the infusion (T = 90 min) (Figure 2A). This alteration in NIRS variables was maintained for the following 120–150 min, i.e. until initial awakening of the animals. In contrast, FTOE was only partially increased during fentanyl administration (Figure 2A).

The median aEEG background activity pattern decreased from 37±12 µV to 20±5 µV after fentanyl bolus administration, which represents a reduction of 41±19% (Figure 2B). This depressed activity remained unchanged during fentanyl infusion and for approximately 120–150 min afterwards. The aEEG activity was partially recovered from T = 210 min to the end of the experiment, reaching levels only 25% lower than the basal values at the last timepoint measured in each individual study. This trend was also applicable to the aEEG amplitudes registered at each timepoint, which followed the same behaviour as the median aEEG amplitude. Indeed, this parameter was significantly reduced after fentanyl bolus (35±12%) (Figure 2B) and remained unchanged until T = 210–240 min, when the differential amplitude began to increase, reaching levels below the basal values by only 12%.

#### Pharmacokinetic variables

**Figure 3:**
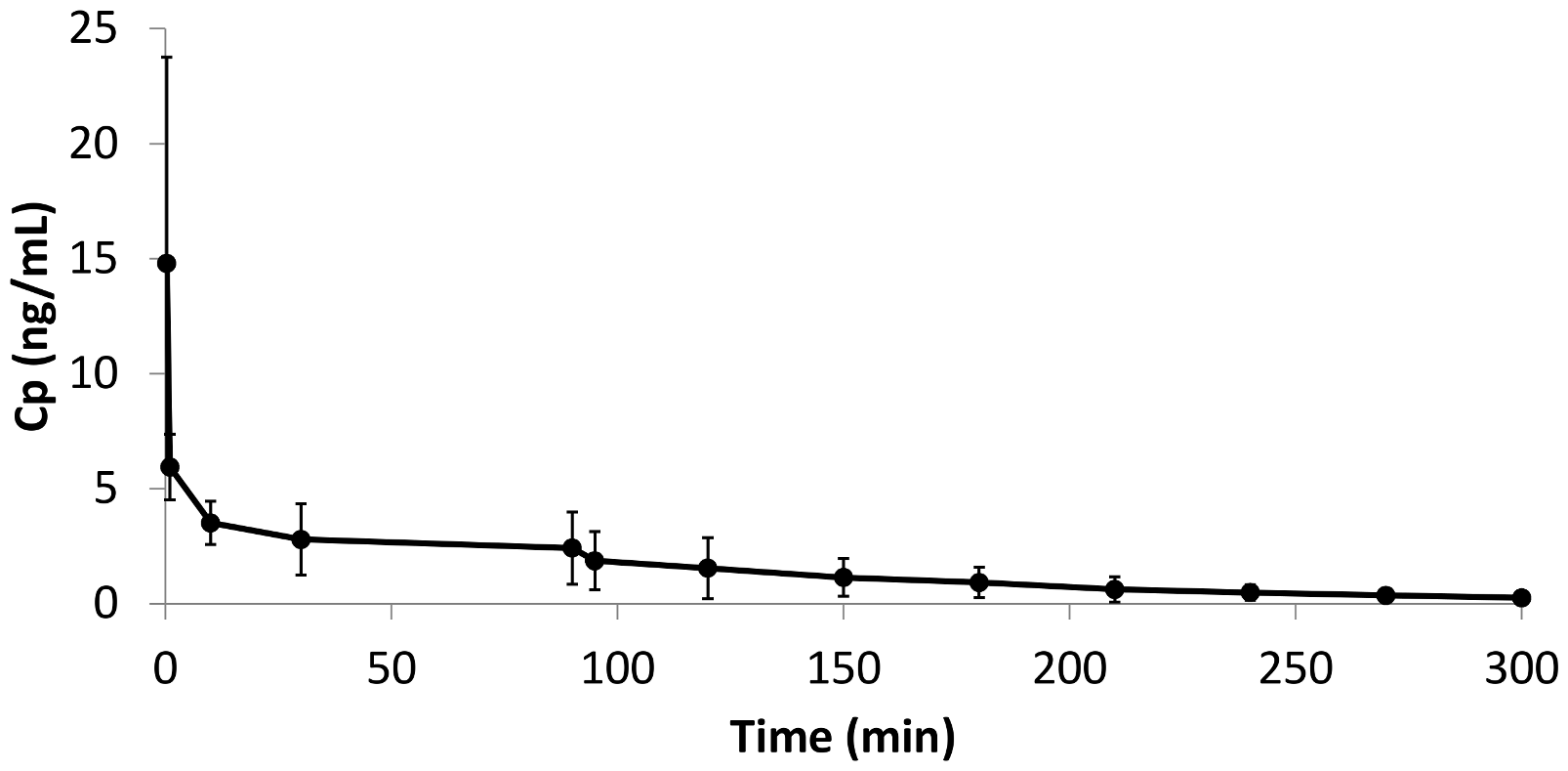
Time course of average fentanyl plasma concentration (Cp) measured in the six piglets’ population after administration of a 5 µg/kg bolus dose immediately followed by a 90 min infusion at a rate of 3 μg/kg/h. Black dots symbolize the mean values for six piglets at each time point, with standard deviations (SD) represented by vertical bars.

The time course of fentanyl plasma concentrations (Cp) measured in the six piglets’ population is shown in Figure 3. The mean fentanyl Cp was 14.8±9.0 ng/ml immediately after bolus administration, 5.9±1.4 ng/ml at 1 min, 3.5±0.9 ng/ml at 10 min, 2.8±1.5 ng/ml at 30 min and 2.4±1.6 ng/ml at 90 min during fentanyl infusion. 5, 30 and 60 min after infusion was stopped, mean Cp was 1.9±1.3, 1.5±1.3 and 1.1±0.8 ng/ml, respectively, which continued decreasing until the last timepoint measured (up to a maximum of 210 min after stopping infusion). Cp at the latest sampling times was mostly very close to the LLOQ of the analytical technique (i.e. 0.2 ng/ml), with solely two piglets showing Cp below this limit at times beyond 150 and 210 min, respectively. Moreover, the fact that plasma fentanyl levels had been cleared to a high extent by the end of experiments (T = 225–300min) is consistent with animals showing initial signs of awakening at this timepoint.

PK variables calculated by NCA are shown in Table 2. As expected, C_max_ corresponded to the sample withdrawn immediately after bolus administration in all cases. Nonetheless, an important IIV was evidenced by these data, given that coefficient of variation (CV) was as high as 60% for C_max_, 56% for plasma concentration-time curve (AUC), and 64% for CL.

**Table 2:** Demographic characteristics and pharmacokinetic (PK) variables of fentanyl calculated for each piglet by noncompartmental analysis (NCA) of the corresponding plasma concentration time curves.

	Sex	BW (kg)	t_max_ (min)	C_max_ (ng/ml)	T_last_ (min)	C_last_ (ng/ml)	AUC_0-t_ (ng*min/ml)	CL (L/min)
**Pig no. 1**	F	1.60	0.10	10.03	300	0.26	319.95	0.021
**Pig no. 2**	M	1.80	0.10	7.77	225	0.68	548.45	0.014
**Pig no. 3**	M	1.76	0.10	16.83	270	BLLOQ	209.75	0.036
**Pig no. 4**	M	1.75	0.10	4.08	225	0.82	518.38	0.012
**Pig no. 5**	F	1.50	0.10	24.44	270	0.68	841.98	0.008
**Pig no. 6**	F	1.97	0.10	25.63	240	BLLOQ	207.44	0.042
**Mean**	.	1.73	0.10	14.80	255	0.43	440.99	0.022
**SD**	.	0.16	0.00	8.96	30	0.33	245.36	0.014
**CV (%)**	.	9.45	0.00	60.56	11.76	77.56	55.64	64.06

BLLOQ: below the lower limit of quantification.

F: Female/ M: Male.

Notwithstanding, calculated CL was 0.022 L/min on average, which is again in line with the value predicted for human neonates (i.e., 0.028 L/min) [7]. Moreover, this is endorsed by the fact that average plasma concentration of fentanyl at the end of infusion tended to be 3 ng/ml as expected, even if a 90 min continuous infusion was not enough for reaching steady state (ss) conditions (Figure 3).

Exploratory analysis revealed that none of the PK variables calculated by NCA was significantly correlated with the duration or depth of the sedative effect, nor with any of the other measured effects.

#### Plasma AAG as a potential source of PK variability

**Figure 4:**
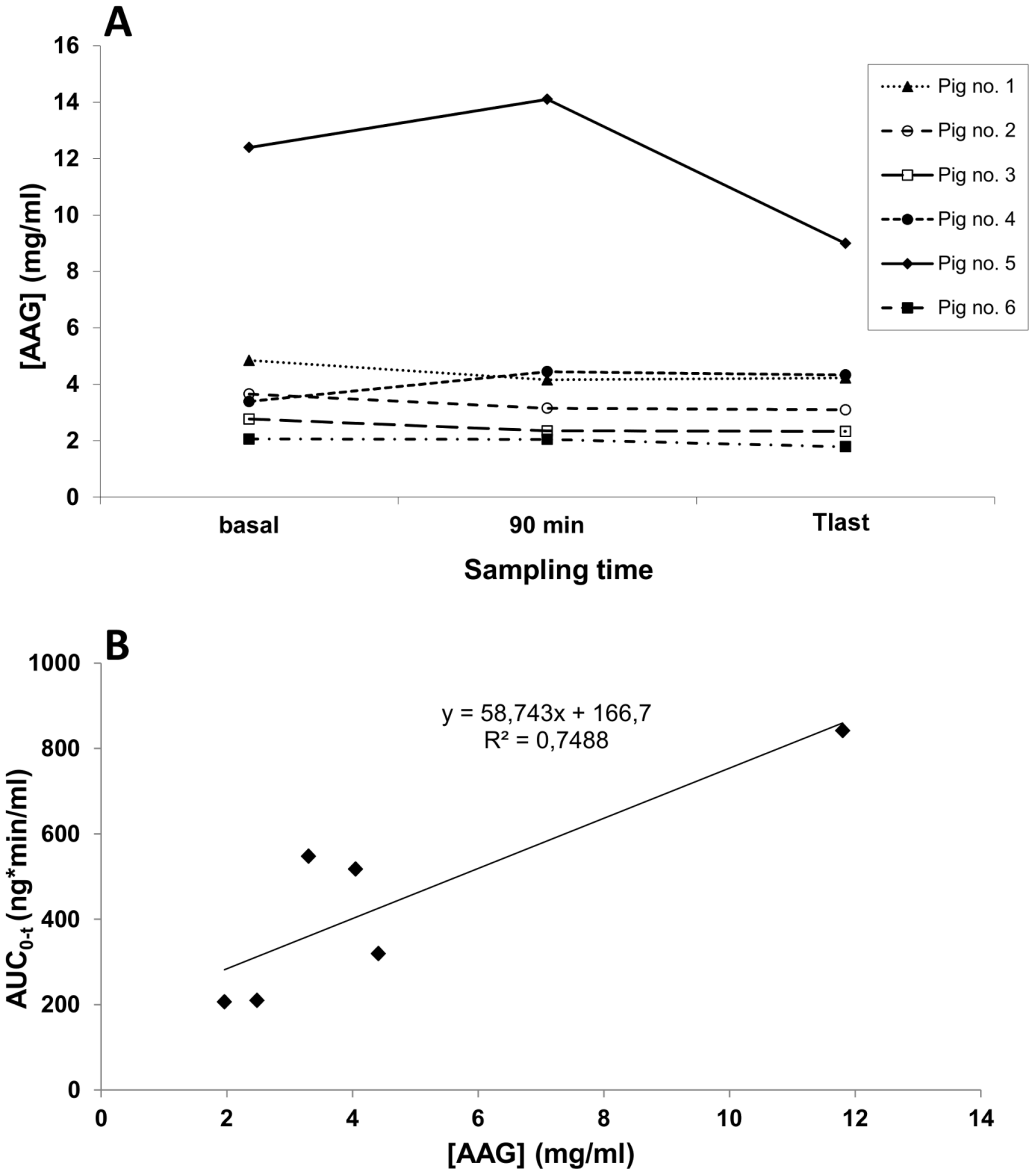
Determination of plasma α_1_-acid glycoprotein (AAG) levels and correlation with the observed interindividual PK variability. A- Individual concentrations of AAG determined by the ELISA method in plasma samples drawn from the six piglets at baseline, T = 90 min and T_last_. B-Correlation between the mean plasma AAG level in each animal (calculated from the measurements at baseline, T = 90min and T_last_) and the corresponding calculated drug exposure (AUC_0_-t).

In most cases, there was no appreciable change on the measured AAG levels between the three plasma samples analyzed within each animal (basal, 90 minute and final) (Figure 4A). Moreover, the absence of significant differences when compared to control animals (data not shown) supports the observation that experimental procedure did not trigger an acute-phase response as represented by elevated plasma AAG levels in these animals, at least within the short period set by the study protocol (i.e., up to 5 hours).

On the other hand, IIV could partially be explained by the differences in measured AAG levels. In this sense, a positive correlation was observed between the mean plasma AAG level in each animal and the corresponding calculated drug exposure (AUC_0-t_), correlation coefficient (R^2^) being 0.749 (Figure 4B).

## Discussion

In clinical practice, pediatric dosing regimens of many medications used in NICU and PICU [3] are often empirically derived from those employed in adults on a body weight basis. However, developmental changes may affect the relationship between dose and exposure (PK) and/or the relationship between exposure and response (PD), which can lead either to an efficacy or safety concern. Development of scientific models predictive of the drug behaviour at the different pediatric stages by considering the changes associated to growth and maturation represents a valid approach towards dosing individualization [7]. In addition or as a complement to theoretical models, the performance of experimental studies in suitable animal species is also a necessary practice. Specifically, the newborn piglet is a representative model for newborn human cardiovascular physiologic development, [13] and also has similar biotransformation pathways to those in humans, including comparable levels and activities for CYP3A4, the isoform responsible for hepatic fentanyl metabolism [14]–[16].

In the present study, the dose of fentanyl administered to newborn piglets (5 µg/kg bolus and 3 µg/kg/h infusion according to the final protocol) is similar to that used in the NICU when fentanyl is given as the sole or principal analgesic agent to neonates with respiratory distress syndrome maintained in mechanical ventilation [17], [20]. The dosing regimen as well as the sampling times and the duration of the study were designed based on the results of a pilot study performed in the first two animals. The systemic drug clearance calculated from this study was 0.022 L/min on average, which is of the same order of magnitude as the one provided by a previously developed ontogeny based model predictive of fentanyl disposition in the newborn (i.e., 0.028 L/min) [7]. Those results not only support the validity of the developed scientific model but also openly endorse, to the best of our knowledge for the very first time, the suitability of the newborn piglet as an experimental model for human neonates concerning fentanyl PK.

Since its development in 1960, it is known that fentanyl and other synthetic opioids may cause skeletal muscle rigidity in neonates and infants after low-dose fentanyl administration [8]–[12]. Similar findings were noted in our newborn piglets in that fentanyl-induced chest wall rigidity occurred almost immediately after the administration of low doses of fentanyl. Accordingly, newborn animals showed a transient alteration of gas exchange, a significantly increased OI and a decreased VEI, corresponding with a simultaneous decrease in C_dyn_ accompanied by the need for increased PIP to maintain an adequate oxygenation and tidal volume.

Robinson and Gregory revolutionized practice after reporting the first use of fentanyl, as the principal anesthetic agent in neonates undergoing ductus ligation surgery [21]. Using HR and MABP responses as an index of adequate anesthesia, these investigators demonstrated that the administration of fentanyl could provide anesthesia with minimal hemodynamic consequences. Subsequent studies also reported hemodynamic stability associated with no change in HR, MABP or CO [22]–[24]. However, changes in MABP without alteration in HR or vice versa have also been observed [25], [26]. In all reported studies, hemodynamic instability is rare, as long as a vagolytic agent (pancuronium and/or atropine) is administered concomitantly.

Usually, in studies carried out in animal models, most cardiovascular variables including HR, MABP and CO, remained unchanged after administration of a wide range of doses [27], [28]. The present findings confirmed stability of CO and MABP after low doses of fentanyl while a progressive increase of HR was observed. This effect had been previously noticed in different experimental models receiving fentanyl, thus suggesting that the observed continuous increase of HR was induced by the drug [2], [29]. Although the mechanism for this is not known, in the absence of more supportive data available, we hypothesized that it may be initially mediated by a withdrawal of vagal tone rather than increased sympathetic output [29]. This hypothesis was based on the unresponsiveness (i.e., no changes in HR, MABP or aEEG) observed in the present study after application of specific painful stimuli at different time intervals from T = 90 min to T = 210 min. However, there was a response to a painful stimulus administered beyond 210 min, coinciding with the initial disappearance of fentanyl effects, which indicated that a sympathetic component may be contributing to maintain elevated HR values in this case.

Besides hemodynamic stability, little is known about the effect of fentanyl in CBF, brain metabolism and aEEG activity in the newborn [2], [28]. In our study, a decrease of tissue hemoglobin index (THI, reflecting changes in cerebral blood volume) (data not shown) and CIO (reflecting changes in CBF), with only a partial increase of FTOE (oxygen consumption) was observed over time as long as the effect of fentanyl was maintained (not response to pain stimuli); thus suggesting that there may be not enough oxygen supply to meet the metabolic requirements of the brain [2], [28]. Moreover, aEEG background activity pattern as well as the aEEG amplitude was diminished during the entire fentanyl effective period. The depressing effect of opioids in aEEG had been previously described in preterm and newborn infants [30]–[33].

Those brain effects returned to basal (pre-infusion) values when the effect of fentanyl ended (response to pain stimuli) suggesting only a transient effect related with fentanyl administration. However, our study was carried out in healthy newborn piglets without any cardiac, hepatic or pulmonary injury, while in clinical practice fentanyl is administered to critically ill newborn infants, who may respond to analgesics in a different manner, given that they usually present hemodynamic parameters already altered at baseline. Thus, more attention should be paid to the effects of sedation and analgesia in sick newborn with impaired cerebral autoregulation, in which fluctuations in CBF and EEG abnormalities could be aggravated by fentanyl administration, even at very low doses. We suggest that the described experimental model could be extremely useful within the purpose of further investigating the impact of fentanyl administration under pathophysiological conditions, as its validity for evaluating respiratory and cardiovascular effects in human neonates has been sufficiently supported by a number of reports.

Otherwise, the observed interindividual PK variability (IIV) seemed not related to sex or body weight, and it may be due to distribution or metabolism related factors. In order to assess the impact of metabolism, further evaluation of individual hepatic blood flow and enzyme activity would be needed, even if maintenance of temperature constant at 37.5–38.5°C throughout the study allowed to preclude variations derived from the strong temperature-dependence of hepatic CYP3A4 activity, as previously shown in juvenile pigs [16].

On the other hand, the mentioned variability could partially be explained by the differences in measured AAG levels, in line with the following pieces of evidence: 1) Fentanyl is highly (80–85%) bound to plasma proteins, primarily AAG, the principal binding protein for basic drugs [5], [6]; 2) Protein binding is one of the determinant factors in drug disposition, known to influence both drug distribution and body clearance, because only unbound drug can transfer among plasma, interstitial fluid and tissue fluid [34], [35]; 3) AAG is one of the most important acute- phase proteins with significant clinical implications, as it becomes elevated in plasma in response to a variety of insults and pathophysiological conditions including surgical interventions, inflammation and stress [36]. Even if the third statement could not be confirmed, at least within the short time frame of this protocol, two first assumptions were supported by the clear correlation observed between AAG plasma levels and drug exposure (AUC_0-t_), which is mainly related to systemic clearance. On the contrary, differences in AAG levels were not sufficient to explain the observed variability in C_max_, most probably because it is influenced by a number of additional factors, not easily measurable in the experimental model, affecting the volume of distribution, as well as by the difficulties linked to accurate extraction of first blood sample immediately after bolus administration. Notwithstanding, elevated plasma peak concentration obtained after the initial bolus dose was not associated with the appearance of adverse effects and rapidly declined (Figure 3) in an exponential manner (i.e., rate of elimination directly proportional to the concentration value), thus having little influence at the latest times, where plasma concentration becomes dependent on the constant rate infusion.

In addition, this study demonstrated the feasibility of maintaining animals adequately controlled solely by fentanyl. This is in line with others reports in the scientific literature, where fentanyl was infused to newborn piglets as the only narcotic agent for as long as 6 h, even if at higher doses [2]. In the present study, Cp <3 ng/ml (the target EC_50_ described for humans newborn) produced adequate degree of sedation in piglets, which may be due to several reasons, including the high observed IIV, the degree of plasma protein binding or a lack of equilibrium between plasma and the site of the effect in the central nervous system (CNS). In this sense, a more comprehensive PK/PD analysis of the data, which is outside the scope of this paper, is needed in order to define a formal concentration-response relationship. Moreover, factors suggested by the present study to have a probable impact on this issue, such as the drug concentration at the site of effect (via sequential sampling from the CNS) and the unbound drug fraction, including the degree of functionality and drug binding affinity for AAG at the neonatal period, should be considered in future experimental protocols.

In conclusion, the attained level of sedation in fentanyl treated newborn piglets was associated with some degree of chest wall rigidity and depressed brain activity, which were only transient as they mostly returned to basal values by the end of experiments. This experimental model is generally regarded as suitable for evaluating respiratory and cardiovascular effects in human neonates, and could therefore be useful to further investigate the impact of fentanyl administration under pathophysiological conditions. Also, fentanyl PK was shown to be comparable between newborn piglets and human neonates, which served to confirm the adequacy of a previously developed predictive model for this age.
